# A Natural Recombinant NADC30‐Like PRRSV Strain in China: Intersection of CH‐1a, QYYZ, and JXA1 Lineages

**DOI:** 10.1155/tbed/6134933

**Published:** 2026-01-05

**Authors:** Heng Zhang, Chengxin Zhang, Yingru Ma, Qin Zhao, Ximei Wang, Qing Pan, Yani Sun

**Affiliations:** ^1^ College of Veterinary Medicine, Northwest A&F University, Yangling, Shanxi, China, nwsuaf.edu.cn; ^2^ Swine Disease R&D Center, Shandong SINDER Technology Co., Ltd., Qingdao, Shandong, China; ^3^ Department of Breast Surgery, Qilu Hospital (Qingdao) of Shandong University, Qingdao, Shandong, China, qiluhospital.com; ^4^ College of Veterinary Medicine, Qingdao Agricultural University, Qingdao, Shandong, China, qau.edu.cn

**Keywords:** CH-1a, JXA1, NADC30-like, porcine reproductive and respiratory syndrome virus, QYYZ, recombinant strain

## Abstract

Porcine reproductive and respiratory syndrome virus (PRRSV) remains a major threat to global swine production. In this study, a novel strain (PRRSV‐AH1) was isolated during a respiratory disease outbreak at a commercial swine operation in Anhui Province, China. Viral replication in MARC‐145 cells was confirmed by observing cytopathic effects (CPEs) and conducting immunofluorescence assays (IFAs). Whole‐genome sequencing revealed a 15,020 bp genome exhibiting 90.0% identity with the NADC30 reference strain, including lineage 1‐characteristic nonstructural polyprotein (Nsp)2 deletions. A distinctive L10S substitution in GP2 aligned with conserved residues of PRRSV‐1. Recombination analysis identified PRRSV‐AH1 as a novel chimera with a NADC30‐like backbone incorporating CH‐1a‐like (lineage 8), JXA1‐like (lineage 8), and QYYZ‐like (lineage 3) sequences—representing the first reported instance of this specific recombination pattern. Experimental infection of piglets induced characteristic PRRSV pathology, including sustained pyrexia, reduced weight gain, prolonged viremia, and neutralizing antibody seroconversion. Comparative pathogenicity analysis revealed that the PRRSV‐AH1 strain elicited febrile responses and peak body temperatures intermediate between classic NADC30‐like strains and JXA1 strains. Notably, PRRSV‐AH1 demonstrated a PRRSV‐N‐specific IgG induction capacity comparable to that of highly pathogenic variants. These findings establish PRRSV‐AH1 as a multilineage recombinant (NADC30‐like, CH‐1a, QYYZ, and JXA1 Lineages) resulting from multiple genetic exchanges, underscoring the increasing complexity of PRRSV diversity in China. Accelerated mutation and recombination across lineages complicate disease control efforts, emphasizing the need for enhanced surveillance, mechanistic recombination studies, and the development of novel vaccines to mitigate future outbreaks.

**Summary**

• A NADC30‐like PRRSV strain isolated in MARC‐145 cells and its full genome sequenced.
• First report of PRRSV recombinant strain involving four genotypes (NADC30‐like, CH‐1a, QYYZ and JXA1 Lineages) in China.
• The recombinant NADC30‐like strain exhibited high pathogenicity in piglets.

## 1. Introduction

Porcine reproductive and respiratory syndrome (PRRS), caused by the PRRS virus (PRRSV), is a highly contagious disease that poses significant challenges to the global swine industry [1]. PRRS primarily causes reproductive failure in sows, characterized by late‐gestation abortions and the farrowing of stillborn, mummified, or nonviable piglets. The virus also induces congenital abnormalities, respiratory dysfunction marked by interstitial pneumonia, and persistent immunosuppression in neonatal swine populations [2, 3].

According to the latest classification by the International Committee on Taxonomy of Viruses, PRRSV belongs to the order Nidovirales and the family Arteriviridae, and is classified into two distinct species: *Betaarterivirus europensis* (PRRSV‐1) and *Betaarterivirus americense* (PRRSV‐2). The prototype strain of PRRSV‐1 is the Lelystad virus, isolated in the Netherlands, while the prototype for PRRSV‐2 is the ATCC VR‐2332 strain, isolated in the United States [4, 5] Although both species cause clinically indistinguishable syndromes in swine [6], they exhibit marked genetic divergence and antigenic disparity, sharing only approximately 60% genome‐wide nucleotide identity and possessing distinct immunodominant epitopes [7]. PRRSV is an enveloped virus with a positive‐sense, single‐stranded RNA genome. It is an approximately 15‐kilobase genome that encodes 10 open reading frames (ORFs): ORF1a, ORF1b, and ORF2a through ORF7. ORF1a and ORF1b direct the synthesis of nonstructural polyproteins (Nsps), which undergo proteolytic processing, while the remaining ORFs encode structural proteins, including GP2a, GP2b, GP3, GP4, GP5, E, M, and N [8]. Among these genomic elements, the Nsp2 and GP5 coding regions exhibit the highest variability, characterized by frequent amino acid substitutions and structural alterations such as localized deletions or insertions [9]. These molecular features make Nsp2 and GP5 valuable markers for tracking PRRSV genetic evolution and key targets for molecular epidemiological investigations [10].

First identified in Europe and North America in 1987, PRRSV has since achieved global distribution [11]. In 1996, PRRSV‐2 was first isolated and identified in China, establishing the prototype strain for subsequent regional spread [12]. Between 2006 and 2016, the highly pathogenic PRRSV (HP‐PRRSV) strain JXA1 emerged as the dominant lineage, characterized by a distinctive 30‐amino‐acid (aa) discontinuous deletion in its Nsp2 coding region [13]. The QYYZ‐like strain, initially identified in Taiwan (China), was shown to have spread to mainland China through phylogenetic evidence in 2010, becoming endemic in southern provinces by 2013 [14]. Since 2017, viral evolution has driven NADC30‐like strains to epidemiological predominance, marked by a 131‐aa discontinuous deletion in Nsp2 compared to classical PRRSV isolates [15, 16]. Concurrently, surveillance data indicate a growing prevalence of NADC34‐like strains within this lineage, which possess a distinct 100‐aa contiguous Nsp2 deletion [17]. The continued emergence of novel NADC30‐like variants reflects frequent recombination among circulating PRRSV strains, underscoring the virus’s adaptive plasticity [18, 19]. In the present study, we characterized a novel natural recombinant PRRSV strain (designated PRRSV‐AH1), derived from CH‐1a, QYYZ, and JXA1 lineages within the NADC30‐like clade, as supported by phylogenetic evidence. We systematically investigated its replication kinetics and cytopathic effects (CPEs) in MARC‐145 cells. Furthermore, the pathogenicity of PRRSV‐AH1 was assessed and compared with the reference JXA1 strain through experimental infection in weaned piglets. Piglets inoculated with PRRSV‐AH1 or JXA1 exhibited typical clinical signs including hyperthermia, lethargy, anorexia, and respiratory symptoms. The JXA1 group, however, induced a more rapid and severe disease course, characterized by an earlier onset of fever, higher peak body temperature, significant weight loss, and more extensive lung lesions at necropsy.

## 2. Materials and Methods

### 2.1. Sample Collection and Processing

In 2022, a suspected PRRSV outbreak occurred on a large‐scale swine farm in Anhui Province with an inventory of 5000 sows, exhibiting approximately 10% morbidity and 7% mortality. To determine the cause of the epidemic, the farm owner submitted five dead 6‐week‐old Yorkshire pigs for inspection. These pigs exhibited purple discoloration on the limbs and blue patches on the skin. We conducted a post‐mortem examination in accordance with laboratory testing procedures and collected damaged lung tissue samples in a biosafety cabinet. Clinical signs included respiratory distress, rough coats, weight loss, and elevated mortality among sows and nursery pigs. All samples were stored at –80°C for preservation.

### 2.2. RNA Extraction and Reverse Transcription

Total RNA was extracted from the lung tissue samples using the AxyPrep Body Fluid Virus DNA/RNA Miniprep Kit (Corning Incorporated). One‐step reverse transcription PCR (RT‐PCR) was performed using the Evo M‐MLV RT‐PCR Kit (Accurate Biology Co., Ltd., Hunan, China). The primers used to amplify the Nsp2 gene were as follows: forward (F): TTTGACTGGGATGTTGTGC; reverse (R): GATGGCTTGAGCTGAGTATTT. The target fragment sizes were approximately 680 bp (NADC30), 980 bp (JXA1), and 1070 bp (VR‐2332). The 50 µL reaction mixture contained 2 µL of total RNA template, 2 µL of one‐step enzyme mix, 25 µL of 2× one‐step reaction solution, 2 µL each of forward and reverse primers, and RNase‐free water to reach the final volume. Cycling conditions were as follows: 50°C for 30 min (reverse transcription), 94°C for 2 min (initial denaturation), followed by 35 cycles of 94°C for 30 s, 49°C for 30 s, and 72°C for 40 s, with a final extension at 72°C for 5 min. Amplified products were analyzed using 1% agarose gel electrophoresis.

### 2.3. Virus Isolation and Identification

PRRSV‐positive samples were homogenized, filtered, and inoculated onto MARC‐145 cell monolayers (the MARC‐145 cell line is preserved and used in our laboratory, and its use has been approved by the Ethics Committee of Northwest A&F University, with the license number 20220301). Blank controls were maintained under identical conditions to monitor nonspecific cellular changes. Cultures were observed for 72–96 h until approximately 80% CPE was evident. The supernatant was collected and passaged onto fresh MARC‐145 cells. This process was repeated for upto five passages. Viral suspensions from each passage were confirmed by RT‐PCR using the methods described above.

### 2.4. Indirect Immunofluorescence Assay (IFA)

At 48 h postinoculation (hpi), PRR SV‐infected MARC‐145 cells were washed three times with phosphate‐buffered saline (PBS; 0.01 mol/L, pH 7.2) after the culture medium was removed. Cells were fixed with 75% methanol for 20 min and washed twice with PBS. PRRSV‐positive pig serum was applied as the primary antibody for 1 h at 37°C, followed by fluorescein isothiocyanate‐conjugated rabbit anti‐pig IgG (Thermo Fisher) as the secondary antibody under identical conditions. After a final PBS wash, fluorescence was visualized using an Olympus inverted microscope. Uninfected MARC‐145 cells served as the negative control.

### 2.5. Viral Growth Curve

Fifth‐passage viral supernatant was inoculated into fresh MARC‐145 cells. Samples were collected at 12‐h intervals over a period of 120 h (10 time points total) and stored at −80°C for downstream analysis. For TCID_50_ determination, serial tenfold dilutions (10^−1^–10^−7^) of the virus suspension in DMEM containing 2% fetal bovine serum were added to MARC‐145 cells seeded in 96‐well plates, with parallel negative controls at each dilution. After 5 days of incubation, CPE was assessed, and TCID_50_ values were calculated using the Reed–Muench method to generate viral growth curves.

### 2.6. Whole‐Genome Sequence Analysis

Viral RNA was extracted from the supernatant of the infected cell culture, and whole‐genome sequencing was conducted by Sangon Biotech (Shanghai) Co., Ltd., using next‐generation sequencing technology. Nucleotide and amino acid homology of the isolate were assessed by comparing its complete genome and individual gene sequences (full‐length genome, Nsp2, and ORF5) with those of representative Chinese PRRSV strains (Table 1). Sequence alignments were performed using the Clustal W algorithm in MegAlign, and phylogenetic trees were constructed using MEGA11 software (neighbor‐joining method, 1000 bootstrap replicates). N‐glycosylation sites in GP5 were predicted using NetNGlyc 1.0 to support the functional characterization of the glycoprotein.

**Table 1:** Information of PRRSV reference strains.

Strain	GenBank number	Origin	Vintage	ORF5‐based genotyping
Lelystad virus	M96262.2	EU	1993	PRRSV 1
CH‐1a	AY032626.1	CN	1996	8
BJ‐4	AF331831.1	CN	1996	5
VR‐2332	AY150564.1	US	1992	5
RespPRRS MLV	AF066183.4	US	1998	5
MLV RespPRRS/Repro	AF159149.1	US	1999	5
TJ	EU860248.1	CN	2006	8
JXA1	EF112445.1	CN	2006	8
HUN4	EF635006.1	CN	2007	8
CH‐1R	EU807840.1	CN	2008	8
NADC30	JN654459.1	US	2008	1
QYYZ	JQ308798.1	CN	2011	3
GM2	JN662424.1	CN	2011	3
JL580	KR706343.1	CN	2013	1
CHsx1401	KP861625.1	CN	2014	1
FJ1402	KX169191.1	CN	2014	1
HENXX‐1	KU950372.1	CN	2014	1
IA 2014 NADC34	MF326985.1	US	2014	1
HNhx	KX766379.1	CN	2016	1
HeN1401	MF766471.1	CN	2018	1
SX2‐1607	MN046241.1	CN	2020	1
HLJWK108–1711	MN046230.1	CN	2020	1
SD	ON254651.1	CN	2022	1
SD‐R	ON254650.1	CN	2022	1
HeN1401	MF766471.1	CN	2018	1

### 2.7. Genetic Recombination Analysis

Potential recombination events between the isolated PRRSV strain and representative reference strains (VR‐2332, JXA1, CH‐1a, NADC30, and QYYZ) were analyzed using RDP4 software. Seven methods were applied, including RDP, GENECONV, BootScan, MaxChi, Chimaera, SiScan, and 3Seq. Recombination events were considered valid only if supported by at least five of the seven methods, each with a significance threshold of *p* < 1 × 10^−6^. Recombination breakpoints were further validated through sliding‐window analysis (200 bp window, 20 bp step size) using SimPlot v3.5.1 with default parameters.

### 2.8. Pathogenicity Analysis

Fifteen clinically healthy, weaned piglets (4–6 weeks old; 5–7 kg body weight), both sexes randomly assigned and of the Yorkshire breed, were obtained from Shandong Sinder Technology Co. (Weifang, China). All animals tested negative for PRRSV antibodies and RNA, as well as for major respiratory pathogens—porcine circovirus type 2 and swine influenza virus—using ELISA (IDEXX 99‐18070) and RT‐qPCR (TOROIVD QPR‐403U kit), respectively. A 7‐day acclimatization period was provided before the experimental procedures to minimize physiological variability caused by stress. All animal experiments were conducted at the biosafety level 2 (BSL‐2) animal facility of Shandong Sinder Technology Co., Ltd., with the animals housed in individual rooms.

The 15 piglets were randomly allocated to three groups (*n* = 5 per group). Group A received 2 mL of PRRSV‐AH1 suspension (1 × 10^5.0^ TCID_50_/mL), Group B with an equal TCID_50_ of PRRSV JXA1 suspension, while Group C (negative controls) was administered an equivalent volume of serum‐free DMEM. All groups were inoculated via intranasal and intramuscular routes, with 1 mL administered via each route. The JXA1 strain (GenBank: EF112445.1) used in this study was obtained from Professor Xiao Shuqi, Lanzhou Veterinary Research Institute, Chinese Academy of Agricultural Sciences. For the challenge experiment, a dose of 2 × 10^5.0^ TCID_50_ was administered, following a standard protocol (trial protocol for inactivated PRRSV vaccine, NVDC‐JXA1 strain). This dose has been internally validated to reliably cause disease in all challenged animals (5/5), consistent with the known pathogenicity of the strain. Clinical parameters—including rectal temperature, respiratory rate, feed intake, and activity—were monitored daily for 21 days postinoculation (dpi). Body weights were recorded every 2 days from 0 to 21 dpi. Jugular venous blood was collected before inoculation (Day 0) and at each weight measurement time point to obtain serum for quantifying viremia and detecting PRRSV‐specific antibodies. Viral RNA in serum was quantified by RT‐qPCR using the TOROIVD QPR‐403U kit with the following primers and probe: F: 5′‐AAGAACTGCATGTCCTGGCG‐3′; R: 5′‐CGACCTCAACCTTACCCCCT‐3′; Probe: 5′‐[FAM]AACTCTATCGTTGGCGGTCGCCCGT[BHQ‐1]‐3′. PRRSV‐specific antibodies in serum were detected using the IDEXX PRRSV Antibody Test Kit, following the manufacturer’s instructions

At 21 dpi, all pigs were euthanized via intravenous injection of sodium pentobarbital at a dose of 150 mg/kg. Lung lesions were observed and photographed. Lung tissues and other major organs were fixed in 4% paraformaldehyde, embedded in paraffin, and sectioned for hematoxylin and eosin staining to evaluate histopathological changes.

The dissected animal carcasses were immediately placed in specialized biohazard bags, sealed, and sterilized in an autoclave. They were then processed by our hazardous waste management partner through high‐temperature incineration. This ensured the complete decomposition of sodium pentobarbital, eliminating any potential for environmental exposure through soil or water systems.

### 2.9. Statistical Analysis

All statistical analyses were conducted using GraphPad Prism version 8.0.2 (San Diego, CA, USA). Intergroup comparisons (Group A vs. Group B) of continuous variables were performed using two‐tailed Student’s *t*‐tests after confirming normal distribution with the Shapiro–Wilk test. Statistical significance was defined as *p* < 0.05.

## 3. Results

### 3.1. PRRSV Detection in Lung Tissues by RT‐PCR

Four out of five lung tissue samples tested positive for PRRSV RNA using RT‐PCR. Amplification with the Nsp2‐specific primer set generated fragments of approximately 680 bp. Sanger sequencing of these amplicons confirmed PRRSV identity, showing 98.7%–99.2% nucleotide homology with reference strains.

### 3.2. Virus Isolation and Identification

MARC‐145 cells inoculated with PRRSV‐positive samples developed progressive CPE, including cell rounding and aggregation, beginning at 72 hpi. By 96 hpi, more than 90% of cells exhibited advanced CPE, characterized by syncytium formation and detachment (Figure 1A). Viral supernatants harvested at approximately 80% CPE tested positive by RT‐PCR targeting the Nsp2 gene, yielding approximately 680 bp amplicon. Sanger sequencing of these amplicons revealed approximately 85% identity with the NADC30 reference strain (GenBank accession no. JN654459.1), confirming successful virus isolation. Indirect IFA demonstrated specific cytoplasmic fluorescence in PRRSV‐infected MARC‐145 cells (Figure 1B), with no signal observed in mock‐infected controls. These findings confirm successful isolation of the strain, which was designated PRRSV‐AH1.

Figure 1Isolation and identification of PRRSV‐AH1. (A) Cytopathic effects in MARC‐145 cells at 0, 72, and 96 h postinoculation with PRRSV‐AH1. (B) Indirect immunofluorescence assay (IFA): PRRSV‐infected MARC‐145 cells at 48 h postinoculation (left) showing specific cytoplasmic fluorescence; uninfected MARC‐145 cells as negative control (right). (C) Growth kinetics of PRRSV‐AH1 as determined by TCID_50_ assay.

**Figure:**
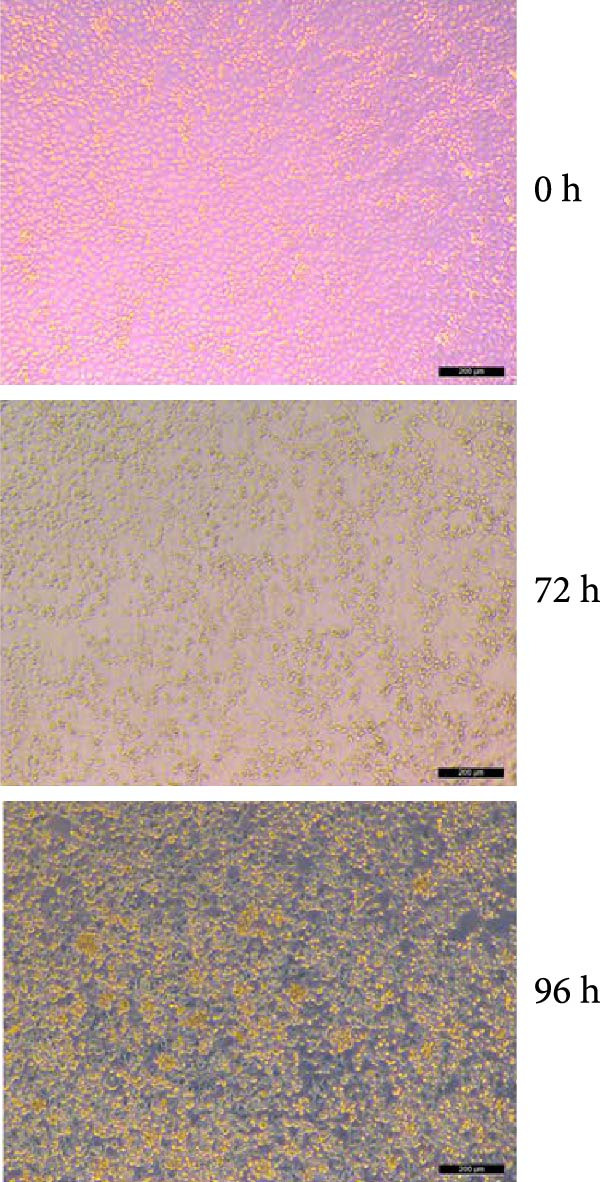
(A)

**Figure:**
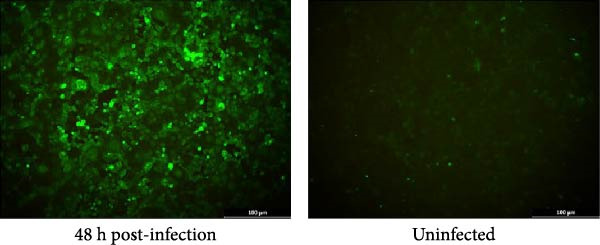
(B)

**Figure:**
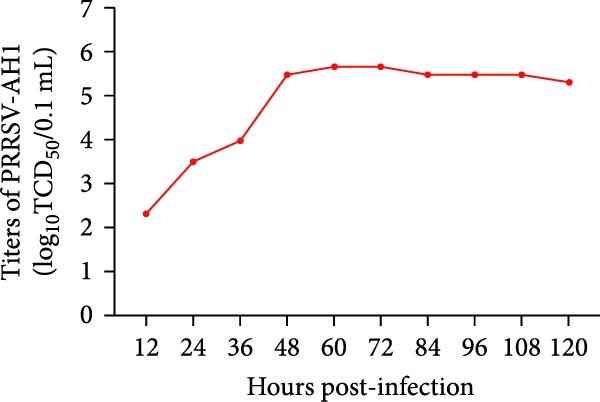
(C)

### 3.3. Growth Kinetics of PRRSV‐AH1 in MARC‐145 Cells

Viral titers were determined by TCID_50_ assay using the Reed–Muench endpoint dilution method. PRRSV‐AH1 exhibited biphasic replication kinetics. Viral titers remained low (10^2.33^ TCID_50_/0.1 mL) during the first 12 hpi and reached a peak (10^5.67^ TCID_50_/0.1 mL) at 48 hpi (Figure 1C). Viral production plateaued between 48 and 120 hpi.

### 3.4. Complete Genome Analysis of PRRSV‐AH1

The complete genome of PRRSV‐AH1 (GenBank accession no. PQ465242) comprises 15,020 nucleotides and exhibits the highest nucleotide identity (90.0%) with the U.S. NADC30 strain. PRRSV‐AH1 shared 86.2%–89.1% identity with other NADC30‐like strains (HNhx, JL580, and HENXX‐1); 84.9%–85.1% identity with classical PRRSV (CH‐1a and CH‐1R) and HP‐PRRSV strains (HuN4, JXA1, and TJ); 84.7% identity with vaccine‐related strain VR‐2332; and 82.2%–83.3% identity with QYYZ and GM recombinant strains. The lowest homology (60.3%) was observed with the European‐type Lelystad virus.

Comparative analysis of PRRSV‐AH1 and reference strains revealed distinct evolutionary patterns across genomic regions. ORF1a, ORF1b, ORF5, ORF6, and ORF7 exhibited the highest nucleotide (88.3%–97.7%) and amino acid identity (88.3%–98.9%) with U.S. NADC30 (GenBank: JN654459.1) and Chinese NADC30‐like variants. Conversely, ORF2 and ORF3 showed highest identity with the QYYZ recombinant strain (nucleotide: 94.2%–94.7%; amino acid: 91.8%–93.3%). ORF4 demonstrated dual high identity with both lineages (nucleotide: 92.0%; amino acid: 93.3%–93.9%), suggesting recombination events within structural gene regions (Figure 2A). Phylogenetic analysis of PRRSV‐AH1 alongside representative global strains was performed using MEGA11 (neighbor‐joining method, 1000 bootstrap replicates). Whole‐genome phylogeny placed PRRSV‐AH1 in lineage 1, forming a monophyletic clade with U.S. NADC30 (JN654459.1) and Chinese NADC30‐like strains JL580 (MF167544) and HNhx (KX766391) (Figure 2B). This cluster exhibited clear divergence from sublineages containing JXA1‐like, VR‐2332‐like, and QYYZ‐like strains, confirming the classification of PRRSV‐AH1 within the NADC30‐like subgroup.

Figure 2Homology and phylogenetic analysis of PRRSV‐AH1. (A) Nucleotide and amino acid sequence similarity between PRRSV‐AH1 and representative PRRSV strains. (B) Whole‐genome phylogenetic tree showing the relationship between PRRSV‐AH1 and major global reference strains.

**Figure:**
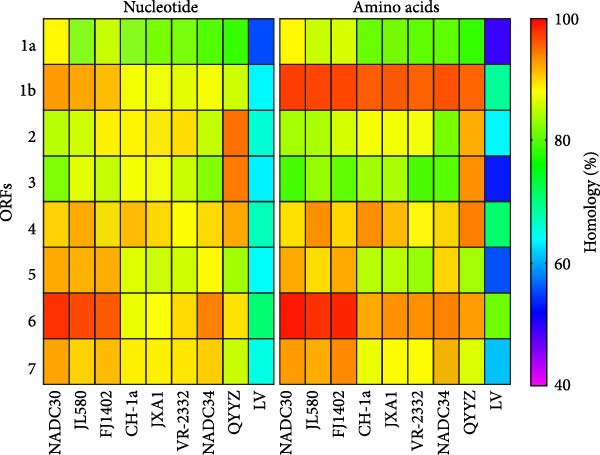
(A)

**Figure:**
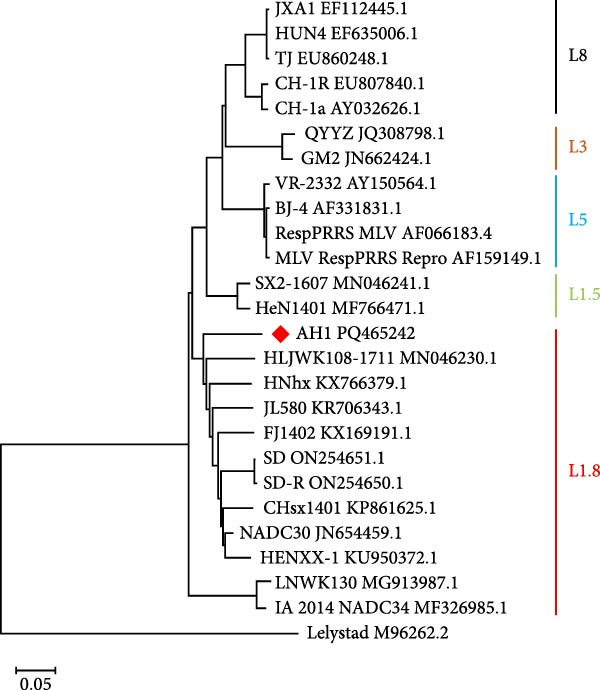
(B)

### 3.5. Amino Acid Variation Analysis in Virulence‐Associated Regions

Comparative residue mapping identified five amino acid substitutions in PRRSV‐AH1 relative to the VR‐2332 strain. Four substitutions—nsp1β‐Ser331Ala, GP3‐Gly83Glu, GP5‐Arg13Gln, and GP5‐Arg151Lys—were consistent with those found in NADC30‐lineage strains. Notably, a unique GP5‐Leu10Ser substitution distinguished PRRSV‐AH1 from all reference strains (including NADC30, HP‐PRRSV, and QYYZ), suggesting the presence of a novel virulence‐associated determinant (Table 2).

**Table 2:** Comparison of virulence related amino acid sites between PRRSV‐AH1 strain and other representative strains.

Protein	AH1	NADC30	JXA1	VR‐2332	QYYZ	MLV RespPRRS/Repro
NSP1β	A331	A331	S331	S331	C331	F331

NSP2	S668	S668	S668	S668	S668	F668
	E821	E821	E922	E952	E950	K952

NSP10	Y949	Y946	Y949	Y946	Y946	H952

GP2	S10	L10	L10	L10	L10	F10

GP3	E83	E83	S83	G83	E83	E83

GP5	Q13	Q13	R13	R13	Q13	Q13
	K151	K151	R151	R151	K151	G151

M	Q16	Q16	Q16	Q16	Q16	E16

### 3.6. Nsp2 Hypervariability and Phylogenetic Relationships

**Figure 3:**
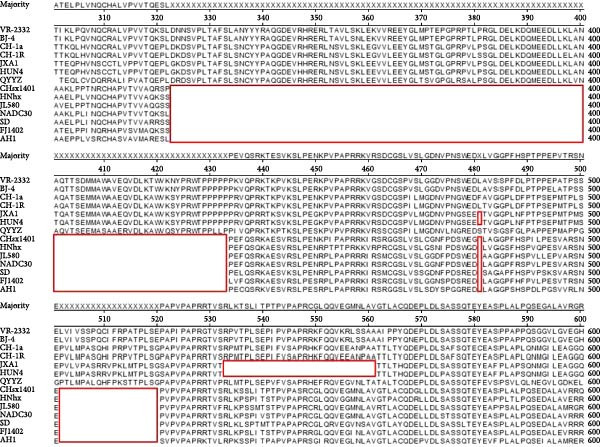
Amino acid sequence alignment of the Nsp2 region. Partial amino acid alignment of the Nsp2 protein in PRRSV‐AH1 compared with representative PRRSV strains, highlighting the 131‐amino‐acid discontinuous deletion.

Alignment of the Nsp2 hypervariable region revealed a characteristic 131‐aa noncontiguous deletion (aa 323–433, 481, and 502–520) in PRRSV‐AH1, consistent with deletions found in Chinese NADC30‐like variants (Figure 3). In contrast, the Nsp2 regions of VR‐2332, JXA1, BJ‐4, and CH‐1a remained conserved. Phylogenetic analysis of this region (neighbor‐joining method, 1000 bootstrap replicates) showed PRRSV‐AH1 clustering with NADC30 (JN654459.1) and contemporary Chinese variants HNhx (KX766391) and CHsx1401 (KY495579), while forming distinct branches from JXA1 (EF112445), QYYZ (JN662424), and VR‐2332 (AY150564) (Figure 4A).

Figure 4Phylogenetic analysis of key genes in PRRSV‐AH1. (A) Phylogenetic tree of the Nsp2 gene from PRRSV‐AH1 and reference strains. (B) Phylogenetic tree of the GP5 gene from PRRSV‐AH1 and representative strains.

**Figure:**
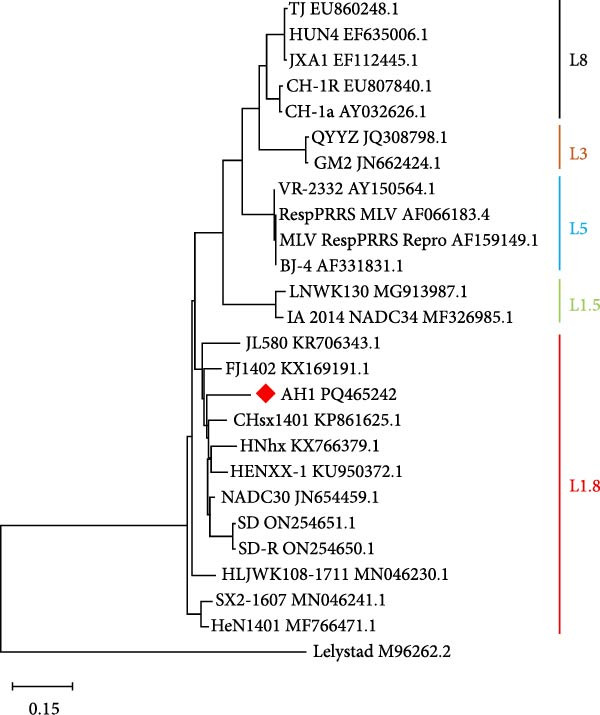
(A)

**Figure:**
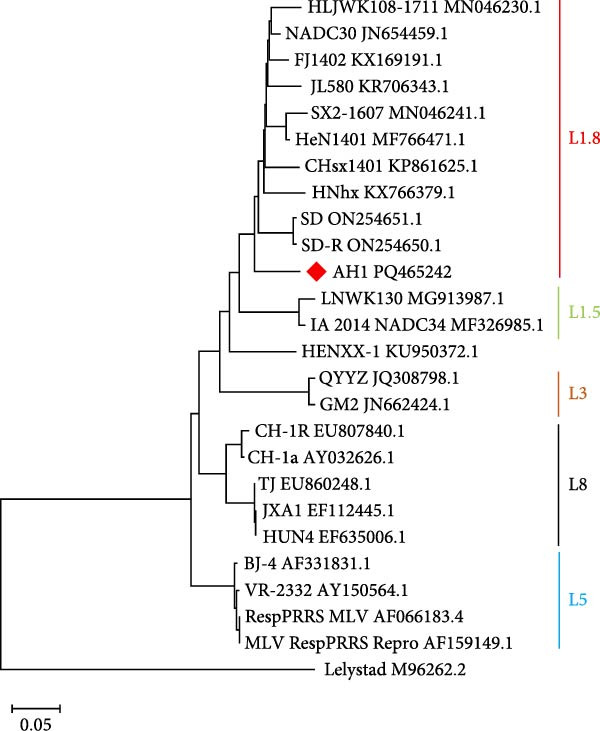
(B)

### 3.7. Lineage‐Defining Features in PRRSV‐AH1 GP5 Protein

Phylogenetic analysis of the ORF5 gene (neighbor‐joining method, 1,000 bootstrap replicates) placed PRRSV‐AH1 within lineage 1, clustering with Chinese NADC30‐like strains JL580 (MF167544) and CHsx1401 (KY495579), while diverging from HP‐PRRSV (JXA1 and EF112445), VR‐2332‐like (VR‐2332 and AY150564), QYYZ‐like (QYYZ and JN662424), and type 1 strains (Figure 4B).

**Figure 5:**
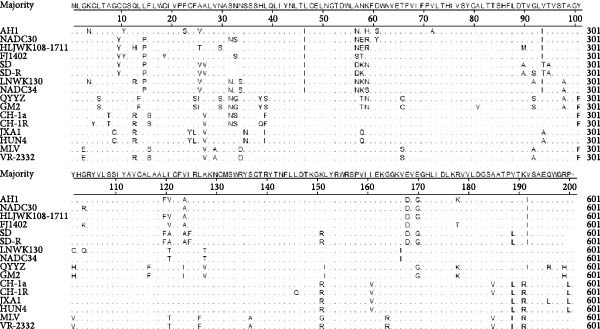
GP5 amino acid alignment and recombination signature. Comparison of GP5 amino acid sequences between PRRSV‐AH1 and reference strains. The alignment highlights conserved and variable regions relevant to virulence and antigenicity.

The GP5 protein of PRRSV‐AH1 exhibited two virulence‐associated substitutions—R13Q and R151K—consistent with those in the NADC30 lineage. At position 137, a known diagnostic site distinguishing wild‐type from vaccine strains, PRRSV‐AH1 retained the wild‐type residue serine (S), identical to JXA1 and NADC30. The neutralizing epitope (residues S37–L45) was fully conserved with MLV RespPRRS/Repro (AY545985), VR‐2332, and NADC30. The decoy epitope (V/A27–N/S30) was also consistent with NADC30 variants and did not exhibit novel mutations (Figure 5). Four N‐glycosylation sites (N30, N34, N44, and N51), known to be essential for viral entry, were identical to those found in the FJ1402 strain (Table 3).

**Table 3:** Comparison of GP5 N‐glycosylation sites between AH1 and other representative strains.

Strain	Potential N‐glycosylation sites (aa)							Quantity
	30–32	33–35	34–36	35–37	44–46	51–53	57–59	
AH1	NAS	NNS	NSS	–	NLT	NGT	—	4
NADC30	–	–	NSS	–	NLT	NGT	—	3
HLJWK108–1711	–	NNS	NSS	–	NLT	NGT	—	4
FJ1402	NAS	–	NSS	–	NLT	NGT	—	4
LNWK130	–	NSS	–	–	NLT	NGT	—	3
JL580	–	–	NSS	–	NLT	NGT	—	3
NADC34	–	NSS	–	–	NLT	NGT	NKS	4
QYYZ	–	–	NSS	–	NLT	NGT	—	3
GM2	–	–	NSS	–	NLT	NGT	—	3
CH‐1a	–	–	NSS	–	NLT	NGT	—	3
CH‐1R	–	–	NSS	–	NLT	NGT	—	3
JXA1	NAS	–	–	NSS	NLT	NGT	—	4
HUN4	NAS	–	–	NSS	NLT	NGT	—	4
MLV RespPRRS/Repro	NAS	NDS	–	–	NLT	NGT	—	4
MLV	NAS	NDS	–	–	NLT	NGT	—	4
VR‐2332	NAS	NDS	–	–	NLT	NGT	—	4

### 3.8. Recombination Patterns Define PRRSV‐AH1 as a Tripartite Recombinant

**Figure 6:**
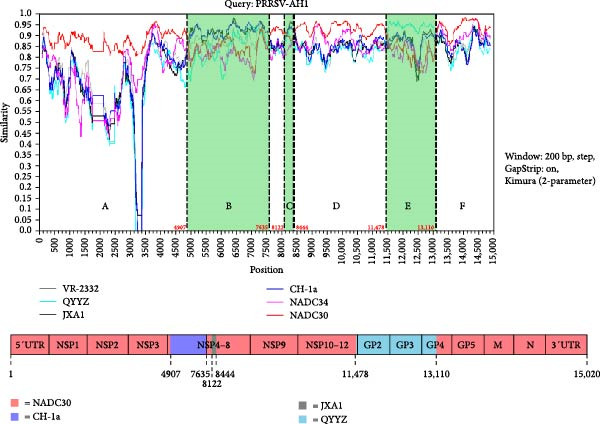
Recombination analysis of PRRSV‐AH1. SimPlot analysis of the full‐genome sequence of PRRSV‐AH1. The strain is identified as a tripartite recombinant with a NADC30‐like backbone (red), and insertion fragments from CH‐1a (blue‐purple; positions 4907–7635 bp), QYYZ (sky blue; 11,478–13,110 bp), and JXA1 (black; 8122–8444 bp).

Whole‐genome analysis identified PRRSV‐AH1 as a tripartite recombinant, derived from NADC30 (lineage 1) with genomic insertions from CH‐1a (positions 4907–7635 bp), QYYZ (11,478–13,110 bp), and JXA1 (8122–8444 bp). These recombination breakpoints were validated by RDP4 software using four or more detection algorithms, each meeting a significance threshold of *p* < 1 × 10^−6^. SimPlot visualization further confirmed the crossover boundaries (Figure 6 and Table 4). Phylogenetic analysis of individual genome regions revealed distinct lineage affiliations. NADC30‐like sequences predominated in ORF1a/b and ORF5*–*7 (lineage 1), while recombinant regions in ORF2*–*4 (lineage 8) and ORF2a (lineage 3) aligned with HP‐PRRSV and QYYZ‐like strains, respectively. This mosaic genome architecture defines PRRSV‐AH1 as a complex recombinant integrating elements from three epidemiologically and genetically divergent PRRSV lineages.

**Table 4:** Possible recombination events between PRRSV‐AH1 and other representative strains in RDP4.

GRE	Major parental	Minor parental	Site /bp	Region	Recombination detection methods and *p*‐values						
					RDP	GENECONV	BootScan	MaxChi	Chimaera	SiScan	3Seq
1	NADC30	CH‐1a	4907–7635	ORF1 (NSP4‐8)	7.7 × 10^−45^	–	2.1 × 10^−44^	5.6 × 10^−24^	4.4 × 10^−28^	7.2 × 10^−19^	8.9 × 10^−61^
2	NADC30	JXA1	8122–8444	ORF1b (NSP9)	7.4 × 10^−11^	2.1 × 10^−07^	2.6 × 10^−11^	6.7 × 10^−3^	1.7 × 10^−3^	2.5 × 10^−2^	–
3	NADC30	QYYZ	11,478–13,110	ORF1b (NSP10‐10) GP2‐3‐4	1.2 × 10^−71^	7.7 × 10^−41^	1.3 × 10^−69^	3.8 × 10^−26^	1.3 × 10^−28^	3.5 × 10^−27^	8.5 × 10^−30^

*Note*: Reorganization positions are based on PRRSV‐AH1 sequences.

### 3.9. Pathogenicity Assessment of PRRSV‐AH1

Pathogenicity evaluation demonstrated that piglets inoculated with the PRRSV‐AH1 strain began to exhibit hyperthermia at 3 dpi, with the mean rectal temperature remaining elevated at 40.5°C from 3 to 18 dpi (Figure 7A). In contrast, piglets in the JXA1 group developed fever as early as 1 dpi, reaching a peak temperature of 41.7°C at 11 dpi, which was significantly higher than that observed in the PRRSV‐AH1 group. From 15 dpi onward, body temperature gradually declined in the JXA1 group, returning to baseline levels (38.5–39.5°C) in most piglets by 19–21 dpi. Meanwhile, piglets in the negative control group maintained normal physiological body temperature throughout the 21‐day observation period.

Figure 7Clinical signs and immune response in piglets. (A) Rectal temperature profiles postinoculation. The green dotted line represents the fever threshold; the red dotted line indicates high fever. (B) Body weight changes. (C) Serum viral load measured by RT‐qPCR. (D) PRRSV‐specific antibody responses (S/P ratios). Lowercase letters indicate differences between group data at the same time point. Data points labeled with the same lowercase letter (e.g., a and a, b and b, c and c) show no significant difference (*p* ≥ 0.05). Data points labeled with different lowercase letters (e.g., a and b, a and c, b and c) indicate a significant difference (*p* < 0.05). If no significant differences are found in all comparisons, the result is marked as ’ns’.

**Figure:**
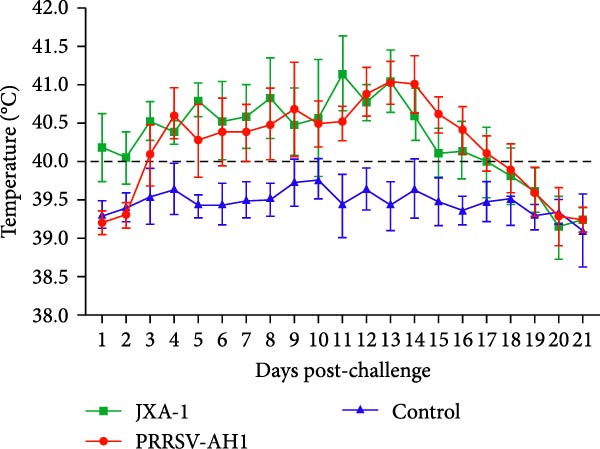
(A)

**Figure:**
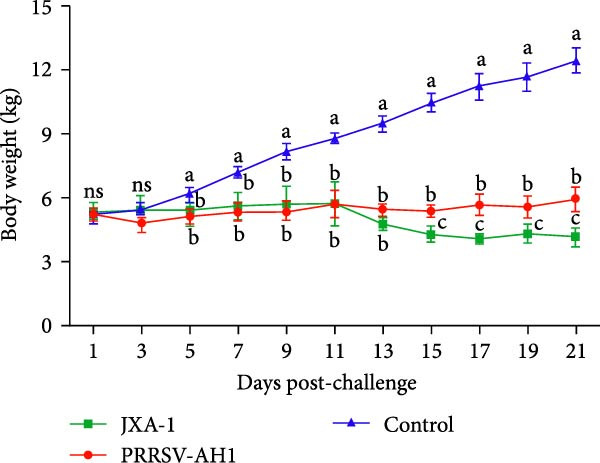
(B)

**Figure:**
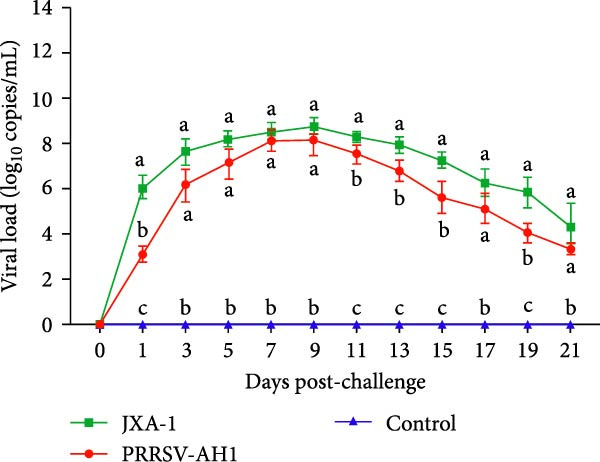
(C)

**Figure:**
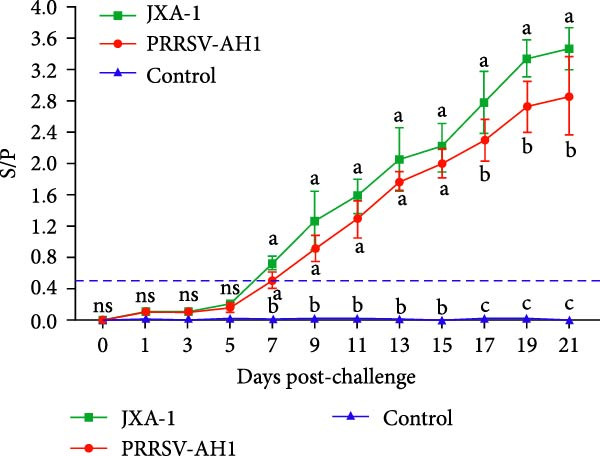
(D)

Clinical signs were similar between the PRRSV‐AH1 and JXA1 groups. In the PRRSV‐AH1 group, two piglets displayed lethargy and anorexia at 3 dpi; by 7 dpi, three piglets showed respiratory symptoms such as coughing, nasal discharge, and shivering; and by 12 dpi, all infected piglets exhibited typical signs of PRRSV infection, including periauricular erythema, tachypnea, and abdominal breathing. In the JXA1 group, four piglets exhibited depression and reduced appetite at 2 dpi, and by 7 dpi, five piglets presented respiratory signs accompanied by periocular edema. No clinical abnormalities were observed in the negative control group throughout the experiment.

Body weight monitoring revealed markedly different growth patterns between the challenged and control groups (Figure 7B). Weight gain was nearly arrested in the challenged groups after inoculation, with the PRRSV‐AH1 group showing an average weight gain of only 0.87 kg and the JXA1 group exhibiting an average loss of 1.1 kg. In contrast, control piglets grew normally, with an average weight gain of 7.36 kg. Statistical analysis indicated that the difference in body weight between the challenged and control groups became significant from 5 dpi (*p* < 0.05), and from 15 dpi onward, the difference between the PRRSV‐AH1 and JXA1 groups also reached statistical significance (*p* < 0.05).

Serological analysis confirmed persistent viremia in both the PRRSV‐AH1 and JXA1 groups. The highest serum viral load, upto 10^9^ copies/mL, was detected between 7 and 9 dpi, and viral RNA remained at high levels throughout the 21‐day observation period. In comparison, RT‐qPCR results were consistently negative in the negative control group (Figure 7C).

Antibody kinetics analysis showed that seroconversion occurred in both PRRSV‐AH1 and JXA1 groups beginning at 5 dpi. By 7 dpi, the sample‐to‐positive (S/P) ratios exceeded the diagnostic threshold of 0.4. Antibody levels increased rapidly thereafter, peaking at 21 dpi with S/P values of 2.96 ± 0.61 and 3.47 ± 0.37, respectively. In the negative control group, S/P ratios remained at baseline levels (0.10 ± 0.05) without notable fluctuation (Figure 7D).

**Figure 8:**
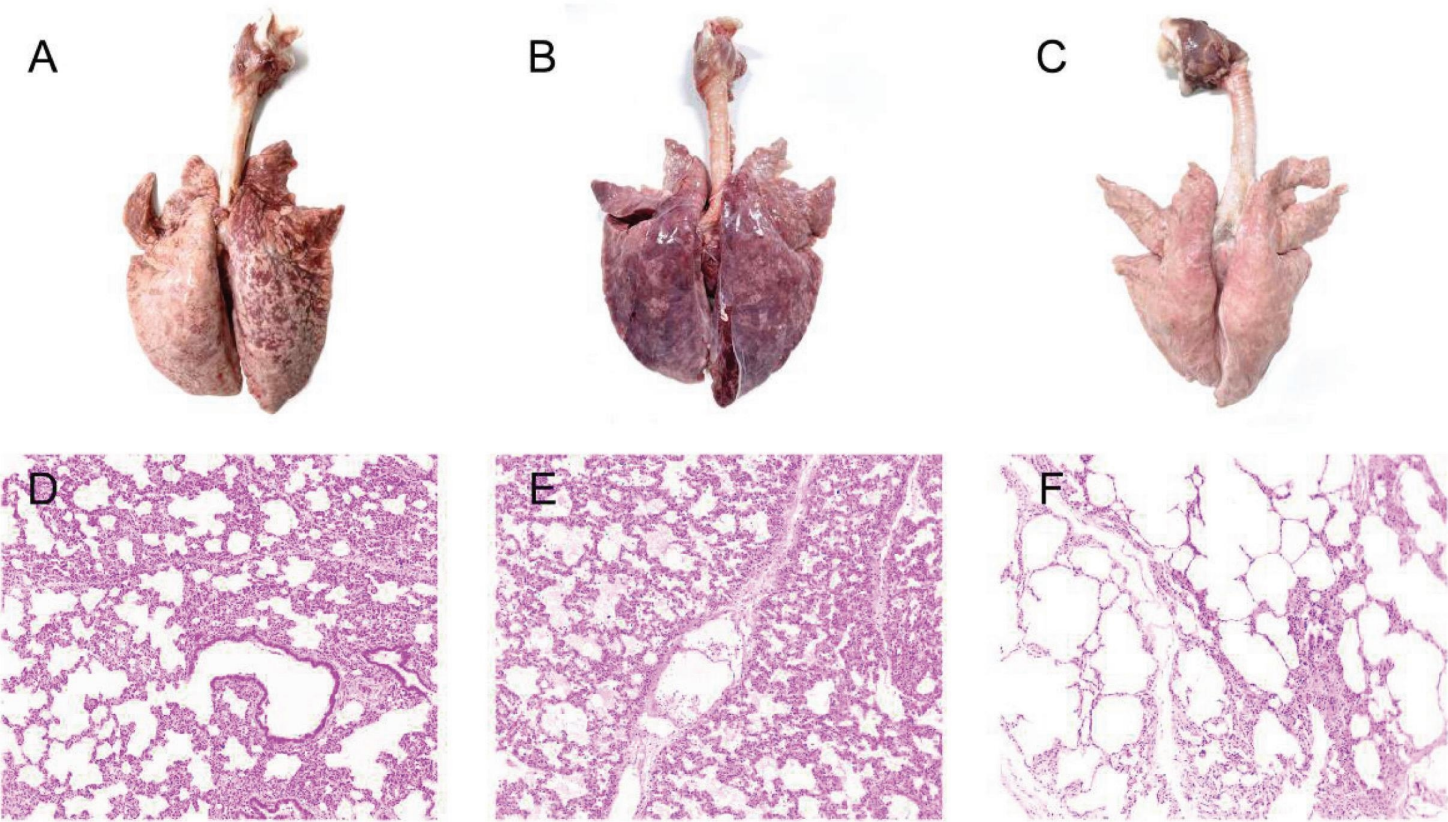
Representative gross lung lesions at necropsy, (A) NADC30 group, (B) JXA‐1 group, and (C) negative control group. Pathological sections of piglet lungs, (D) NADC30 group, (E) JXA‐1 group, and (F) negative control group.

Necropsy of piglets from the PRRSV‐AH1 (Figure 8A) and JXA1 (Figure 8B) groups revealed severe pulmonary lesions. Lungs from the PRRSV‐AH1 group showed multifocal consolidation with a mottled appearance, reduced elasticity and glossiness, and approximately 90% of the lung tissue was affected with significant parenchymal degeneration. The JXA1 group exhibited darker lungs with diffuse lesions, more severe inflammation, and more extensive parenchymal degeneration. The lungs of the negative control group showed no significant lesions (Figure 8C). Histopathological examination indicated inflammatory cell infiltration in the alveolar septa and structural disruption of alveoli with severe inflammatory exudate in the PRRSV‐AH1 group (Figure 8D). The JXA1 group displayed markedly thickened alveolar septa, prominent inflammatory cell infiltration, collapsed alveolar spaces filled with exudate and hyperplastic cells, and considerable interstitial pathology, indicating more severe lung injury (Figure 8E). In contrast, lungs from the negative control group maintained clear alveolar structure without significant pathological damage (Figure 8F).

## 4. Discussion

Since its initial identification in the United States in the 1980s, PRRSV has remained a significant challenge to the global swine industry. The emergence of NADC30‐like strains in China in 2013, in particular, has been closely associated with high nucleotide substitution rates and frequent recombination events involving multiple PRRSV‐2 sublineages [13, 18–20]. Recombination is now recognized as a primary mechanism driving PRRSV genetic evolution. Currently, the predominant PRRSV strains circulating in China can be classified into four major lineages: lineage 1 (NADC30‐like/NADC34‐like), lineage 3 (QYYZ‐like), lineage 5 (VR‐2332‐like), and lineage 8 (JXA1‐like/CH‐1a‐like) [21, 22]. The genetic diversity of field strains, accumulation of cross‐lineage mutations, and ongoing recombination have collectively contributed to the emergence of novel strains with enhanced virulence in China [18, 23]. These genetic changes have led to a significant decline in the cross‐protective efficacy of existing vaccines against prevalent NADC30‐like strains [24–27].

In this study, we successfully isolated an NADC30‐like PRRSV strain, designated PRRSV‐AH1, from a large‐scale pig farm in Anhui Province experiencing a suspected PRRS outbreak. The strain exhibited the characteristic discontinuous deletion of 131 amino acids in the hypervariable region of the Nsp2 gene, a hallmark of NADC30‐like strains. Phylogenetic analysis confirmed its clustering with prevalent Chinese NADC30‐like strains, including JL580 and HNhx. Whole‐genome recombination analysis identified PRRSV‐AH1 as a novel natural recombinant with an innovative recombination pattern.

The recombination pattern of PRRSV‐AH1 exhibits several innovative characteristics. Unlike previously reported simple biparental recombination events, this strain utilizes a lineage 1 (NADC30‐like) backbone while incorporating genomic fragments from three distinct lineages: specific regions show insertions from lineage 3 (QYYZ‐like) and lineage 8 (CH‐1a‐like and JXA1‐like). More remarkably, this multilineage integration occurs within a relatively compact genomic scope, where six recombination breakpoints are precisely grouped into three distinct functional regions encompassing both nonstructural and structural protein coding areas. This precise multilineage integration pattern is exceptionally rare among currently documented PRRSV recombinants and may represent a novel viral evolutionary strategy.

This finding corroborates recent domestic studies indicating that NADC30‐like strains have become core parents driving PRRSV genetic diversity and the emergence of novel recombinant strains. The unique recombination pattern of PRRSV‐AH1 further expands our understanding of PRRSV recombination capacity—demonstrating that the virus can achieve precise sequence exchange among multiple lineages under natural conditions, beyond traditional biparental recombination. Research shows that NADC30‐like strains in China exhibit extensive interlineage recombination capability, forming various recombinant combinations involving two to three lineages (e.g., lineages 1, 3, 8, and 1.5) [28–30]. However, PRRSV‐AH1 demonstrates unique preferences in recombination breakpoint selection, with its breakpoint distribution covering both common nonstructural protein coding regions (such as Nsp9) and less frequently reported structural protein areas. For instance, compared to the seven breakpoints identified in strain HN‐YL1711 [28], PRRSV‐AH1 achieves more diverse lineage integration through fewer breakpoints. This “high‐efficiency” recombination pattern may provide the virus with significant evolutionary advantages.

Notably, the identified substitution at position 10 in the GP2 protein, where leucine (L10) is replaced by serine (S10), may be associated with this special recombination pattern. This mutation aligns with conserved residues in PRRSV‐1 GP2 proteins. Such cross‐species convergent evolution in structural protein regions may directly reflect the impact of multiple recombination events on viral protein function.

Recombination events maybe influence the pathogenicity of NADC30‐like strains. In this study, PRRSV‐AH1 induced moderately severe clinical signs in piglets, including high fever and PRRSV‐N‐specific antibody production, with pathogenicity intermediate between typical NADC30‐like strains and highly pathogenic HP‐PRRSV. This unique pathogenic phenotype likely relates directly to its innovative recombination pattern—the integration of multiple lineage fragments may produce synergistic effects, enabling the virus to acquire pathogenic capabilities surpassing those of simple recombinant strains. This observation aligns with literature reports that recombination events, particularly when incorporating key virulence‐related fragments from HP‐PRRSV (lineage 8), can significantly enhance viral pathogenicity. For example, strain GD‐7, through recombination with HP‐PRRSV TJ‐like fragments, caused high mortality in piglets and pregnant sows [31]; while strain SCABTC‐202305, which uses HP‐PRRSV as its primary parent, showed significantly higher pathogenicity than its NADC30‐like parent‐derived counterparts [32].

However, the exact mechanisms underlying PRRSV gene recombination remain unclear, and recombination breakpoints between different viral strains appear to occur randomly [33, 34]. The precise multiple recombination pattern exhibited by PRRSV‐AH1 challenges conventional random recombination theories, suggesting the existence of yet unelucidated recombination hotspot preference mechanisms. Furthermore, recombination introduces changes in viral biological characteristics and challenges existing control strategies. Some recombinant strains (e.g., HN‐YL1711) exhibit altered cell tropism due to recombination in specific regions, losing infectivity for conventional MARC‐145 cells [28]; while antigenic changes resulting from recombination (such as amino acid mutations in key neutralizing epitopes of the GP5 protein) may enable strains to escape vaccine‐induced immune protection [30–32].

The high mutation rate of PRRSV, combined with its capacity for inter‐lineage recombination in pig farms, presents a major obstacle to disease control. The emergence of PRRSV‐AH1’s innovative recombination pattern indicates that PRRSV evolution may be progressing toward more complex and precise directions. Future research should prioritize: (a) establishing systematic PRRSV surveillance networks with particular focus on strains exhibiting complex recombination patterns; (b) elucidating the molecular mechanisms of recombination between different PRRSV lineages, especially the formation of multiple recombination events; and (c) developing and deploying novel vaccines specifically targeting recombinant PRRSV strains. Through continuous genomic monitoring of recombination events, clarification of complex recombination pattern formation mechanisms, and development of new‐generation vaccines based on prevalent recombinant strains, we can effectively control PRRS spread in China.

## Ethics Statement

The animal study was reviewed and approved by the Animal Care and Ethics Committee of Shandong SINDER Technology Co., Ltd., (Approval Number: 2024030102). All procedures were performed in accordance with the National Institutes of Health Guide for the Care and Use of Laboratory Animals, and every effort was made to minimize the suffering and number of animals used.

## Disclosure

All the authors have contributed to the manuscript and approved the submitted version.

## Conflicts of Interest

The authors declare no conflicts of interest.

## Author Contributions

**Heng Zhang and Chengxin Zhang**: software, data curation. **Heng Zhang**, **Chengxin Zhang, and Yingru Ma**: formal analysis, methodology. **Heng Zhang and Qin Zhao**: project administration. **Yani Sun and Qin Zhao**: supervision. **Heng Zhang and Chengxin Zhang**: writing – original draft. **Yani Sun**, **Ximei Wang**, **Heng Zhang**, **Qing Pan**, **and Qin Zhao**: writing – review and editing. Heng Zhang and Chengxin Zhang have contributed equally to this study.

## Funding

The authors declare that financial support was received for the research, authorship, and/or publication of this article. This work was supported by the Major Scientific and Technological Innovation Project (MSTIP) (Grant 2023CXGC010705).

## Data Availability

The data that support the findings of this study are available upon reasonable request. The data are not publicly available due to ethical considerations.
